# Evaluation of TAM Receptor Targeting in Pathophysiology of Idiopathic Pulmonary Fibrosis

**DOI:** 10.3390/medicina61101837

**Published:** 2025-10-14

**Authors:** Nicole Vercellino, Luciana L. Ferreira, Elisa Zoppis, Alice Di Tizio, Zohre Sabihi Ahvaz, Rosalba Minisini, Francesco Gavelli, Pier Paolo Sainaghi, Filippo Patrucco, Mattia Bellan

**Affiliations:** 1 Department of Translational Medicine, University of Piemonte Orientale, 28100 Novara, Italy; 2 Department of Health Sciences, University of Piemonte Orientale, 28100 Novara, Italy; 3 Department of Cardiac, Thoracic, Transplantation and Vascular Surgery (HTTG), Hannover Medical School (MHH), 30625 Hannover, Germany; 4 Emergency Medicine Department, Azienda Ospedaliero-Universitaria, Maggiore della Carità, 28100 Novara, Italy; 5 Center for Autoimmune and Allergic Disease (CAAD), University of Piemonte Orientale, 28100 Novara, Italy; 6 Division of Internal Medicine and Rheumatology, Medcial Department, Maggiore della Carità Hospital, 28100 Novara, Italy; 7 Division of Respiratory Diseases, Medical Department, Maggiore della Carità University Hospital, 28100 Novara, Italy

**Keywords:** Idiopathic Pulmonary Fibrosis (IPF), TAM receptors, Gas6, fibrosis, Bemcentinib, R428, LDC1267, nintedanib, therapeutic approaches

## Abstract

*Background and Objectives*: TAM receptors—Tyro3, Axl, and Mer—and their ligand Growth Arrest-Specific 6 (Gas6) represent a pleiotropic system implicated in fibrosis. Increased Gas6 and Axl expression have previously been observed in lung samples and fibroblast cultures from Idiopathic Pulmonary Fibrosis (IPF) patients. The study explored the contribution of Gas6/TAM system in fibrosis development and the impact of its pharmacological inhibition in fibroblasts. *Materials and Methods*: IPF fibroblasts (IPF FBs) and control human pulmonary fibroblasts (HPFs) were treated with R428 (Axl-specific inhibitor), LDC1267 (TAM inhibitor), or Nintedanib (an IPF-approved drug) to evaluate the influence of these drugs on cell proliferation, migration, and the expression of pro-inflammatory and pro-fibrotic genes. Fibroblast-to-myofibroblast differentiation was induced by TGF-β. The impact of IPF FBs and HPF on macrophage polarization was investigated through a co-culture of fibroblasts with monocyte-derived macrophages, with the further gene expression analysis of markers of the M1 (pro-inflammatory) or M2 (pro-fibrotic) polarization forms. *Results*: Cell proliferation was monitored in fibroblasts treated with TGF-β, the drugs, and their combination. In the presence of LDC1267 and Nintedanib, minor differences in cell confluence were detected between IPF FBs and HPFs; R428 (1 μM) seemed to have a higher inhibitory impact on IPF FBs. Regarding cell migration, the fibroblasts treated with LDC1267 exhibited slower wound closure. R428 treatment led to a relative wound closure of 76% in HPFs but only 56% in IPF FBs (60 h). R428 (1 μM) significantly reduced the expression of the pro-fibrotic markers ACTA2, COL1A1, and FN1 in HPFs and IPF FBs compared to TGF-β treatment. HPFs and IPF FBs co-cultured with monocyte-derived macrophages demonstrated a significantly increased expression of MRC1 while the expression of FN1, TNFα, and CXCL10 was moderately increased. *Conclusions*: These findings suggest that R428 and LDC1267 modulate the proliferation, migration, and gene expression of activated fibroblasts via TAM signaling. Fibroblast-mediated effects on macrophage polarization underscore the relevance of intercellular crosstalk in fibrotic disease.

## 1. Introduction

Interstitial lung diseases (ILDs) include a group of diffuse parenchymal infiltrative lung disorders classified according to etiologic, clinical, radiologic, and histopathologic features [1]. Idiopathic Pulmonary Fibrosis (IPF) is one of the most common forms of ILD [2] of unknown etiology as suggested by its denomination “idiopathic” [3]. IPF is the prototype of Idiopathic Interstitial Pneumonias (IIPs); its signature is a specific histopathological pattern characterized by the presence of usual interstitial pneumonia (UIP) [4]. IPF is a chronic lung disease that causes the progressive fibrosis of the lung tissue, causing a progressive worsening of dyspnea and cough, which exert a significant impact on patients’ quality of life [5]. The disease progression, as well as the fibrosis, thickens the alveoli, making it increasingly difficult for oxygen to enter the bloodstream. This condition leads to breathing difficulties, a decline in lung function, and, in many cases, respiratory failure [6].

IPF is a multifactorial condition that is probably the result of the interaction among genetic, immunological factors and environmental exposures, ultimately leading to fibrogenesis [7].

Type 2 alveolar epithelial cells (AEC2s) are stem cells that are involved in the renewal of type 1 alveolar epithelial cells (AEC1s) during homeostasis or after lung injury. The activity of AEC2s has been found to be altered in patients affected by IPF [8]. During EMT-induced damage, molecular alterations in alveolar epithelial cells trigger the expression and release of pro-fibrotic factors such as cytokines and growth factors, which promote the migration, proliferation, and differentiation of fibroblasts into myofibroblasts, ultimately resulting in extracellular matrix accumulation [9,10] and lung fibrosis [11,12,13]. Moreover, these mediators, involved in fibroblast-to-myofibroblast transition, are also implicated in the formation of a typical hallmark of IPF, the so-called fibroblastic foci (FF); these clusters of active fibroblasts and myofibroblasts are located near to hyperplastic AECs contributing to mucopolysaccharides and collagen production [14,15].

Nintedanib is a receptor blocker for multiple tyrosine kinases that plays a role in the production of fibrogenic growth factors such as platelet-derived growth factor, vascular endothelial growth factor (VEGF), and fibroblast growth factor (FGF). It slows down the progression of IPF [16]. In two consecutive trials (INPULSIS-1 and INPULSIS-2), a total of 1066 patients with IPF were randomly assigned to receive Nintedanib 150 mg or placebo twice daily for 52 weeks [17]. In INPULSIS-1, the Nintedanib group showed a lower annual decline in forced vital capacity (FVC) compared to the placebo group, with a difference of 125.3 mL/year; similar results were observed in INPULSIS-2,in which the FVC decline difference was 93.7 mL/year. In INPULSIS-1, there was no significant difference in the time to first exacerbation between Nintedanib and placebo, while in INPULSIS-2, there was an increase in the time to first exacerbation.

TAM receptors—Tyro3, Axl, and Mer—belong to the subfamily of tyrosine kinase receptors (RTKs) [18]. These receptors are activated when they bind to their ligands, which are Growth Arrest-Specific 6 (Gas6) and protein S (ProS1) [19,20,21]. Moreover, TAM receptors can be found in human plasma in soluble form (sTyro3, sAxl, and sMer) as a result of membrane receptor cleavage. Gas6 and TAMs represent a highly pleiotropic system that is implied in many important biological functions. They are involved in physiological processes such as cell growth, survival, differentiation, motility, and adhesion [22], as well as in maintaining the homeostasis through the regulation of immune, nervous, vascular, and reproductive functions [23]. Notably, this system might impair the apoptotic bodies phagocytosis implied in the autoimmunity processes [24], and it is also particularly important in the regulation of inflammation and in the development of fibrosis [25].

Recently, an increased expression of Gas6 and Axl has been observed in lung samples and in fibroblast culture from patients affected by IPF compared with normal subjects [26].

Therapeutic strategies targeting the Gas6/TAM axis, through anti-Gas6 or anti-Axl antibodies or small-molecule inhibitors, have been shown to modulate fibroblast-to-myofibroblast activation in IPF. Moreover, both Gas6 deficiency and TAM inhibition attenuated pulmonary fibrosis in murine models [26,27].

These findings underscore the active involvement of the Gas6/TAM axis in IPF pathogenesis and support the further investigation of Gas6/Axl inhibition as a potential therapeutic strategy.

Based on this, the aim of the study was to explore the involvement of Gas6/TAM in fibrotic signaling and to evaluate the inhibition of this system in fibroblast activity.

## 2. Materials and Methods

### 2.1. Cell Culture

IPF fibroblasts (IPF FBs CCL-134, ATCC) were cultured in Ham’s F12K medium supplemented with 10% FBS and 1% penicillin/streptomycin solution, and cells were used between passage 11 and 24. Control human pulmonary fibroblasts (HPFs, C12360, PromoCell, St. Louis, MO, USA) were cultured in 25 mM glucose DMEM supplemented with 10% FBS and 1% penicillin/streptomycin solution, and cells were used between passages 7 and 15. The cell culture was maintained at 37 °C in a humidified atmosphere of 5% CO_2_. When IPF FBs and HPFs reached approximately 80% confluence, the cells were split, and the medium was changed every 3–4 days.

**Figure 1:**
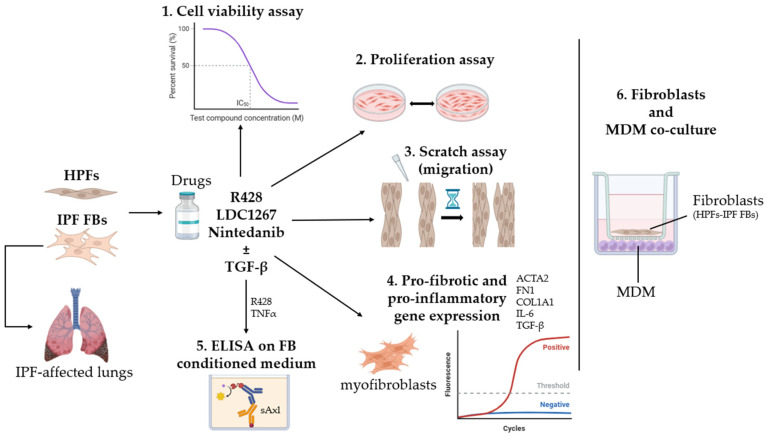
Schematic representation of the study experimental design.

The schematic representation of the study experimental design is reported in Figure 1.

### 2.2. MTT Assay: Metabolic Cell Viability

Cells were seeded at a density of 5000 cells/cm^2^ in 96-well plates. After 24 h, the medium was replaced by medium containing the drugs at different concentrations (R428, LDC1267 or Nintedanib) or the vehicle (DMSO). After 48 h, the medium containing drugs/vehicle was removed from the plate, and 100 μL of MTT solution (1 mg/mL in medium without FBS) was added to each well, and the plate was incubated for three hours. The MTT solution was removed and 100 μL DMSO was added to each well to dissolve the formazan crystals. After proper mixing, 50 μL of the solution was transferred to another plate, and optical density was read at 570 nm through Victor X4 spectrophotometer (PerkinElmer, Waltham, MA, USA).

### 2.3. Cell Proliferation

For the analysis of fibroblast proliferation, 5000 cells/cm^2^ were plated in a 96-well plate. After 24 h, the media were changed to media containing different concentrations of the drug treatments, which included R428 (0.05 μM, 0.1 μM, and 1 μM), LDC1267 (1 and 5 μM), or Nintedanib (0.1 and 1 μM) alone or in combination with TGF-β (10 ng/mL). The plate was placed on IncuCyte^®^ (Sartorius, Göttingen, Germany) and imaged every 6 h during 132 h at 10× magnification. Phase Object Confluence was quantified using IncuCyte^®^ software (Sartorius Group, v2025A, Göttingen, Germany).

### 2.4. Wound Healing Scratch Assay

The effects of TGF-β and the drugs on fibroblast wound closure were evaluated through a wound healing scratch assay. The fibroblasts were seeded in 96-well plates at a density of 10,000 cells/cm^2^. When cultures reached 100% confluence (3–4 days later), a scratch was created in the cell monolayer using the IncuCyte^®^ (Sartorius) wound maker tool. After washing with PBS, the fibroblasts were subsequently incubated with TGF-β alone or in combination with R428 (0.1 and 1 μM), LDC1267 (1 and 5 μM), and Nintedanib (0.1 and 1 μM) in media containing 1% FBS to reduce the effects of fibroblast proliferation on wound closure. Image acquisition was performed every 4 h for a total duration of 60 h using the IncuCyte^®^ (Sartorius) live cell imaging system at 10× magnification. For each time-point, Relative Wound Density (%) was calculated using the Scratch Wound analysis of the IncuCyte^®^ software.

### 2.5. RNA Extraction, Reverse Transcription, and RT-qPCR

Total RNA was extracted from 6-well plates using Nucleozol (Cat. #740404.200, Macherey-Nagel, Düren, Germany) according to the manufacturer’s instructions. After RNA quantification (NanoDrop™ One/OneC Microvolume UV-Vis Spectrophotometer, Thermo Scientific™, Waltham, MA, USA), RNA was retrotranscribed into cDNA using High Capacity cDNA Reverse Transcription Kit (#4368814). Expression levels were quantified by quantitative real-time-qPCR using PowerUp™ SYBR™ Green Master Mix (#A25742) (Applied Biosystems™, Foster City, CA, USA), 10 ng cDNA, and 500 nM of each primer. No template control (NTC) and no reverse transcriptase control (NRT) were included in all the running plates. Reaction efficiency was determined based on serial dilutions of a randomly selected sample. HPRT and/or TBP were used as housekeeping genes. The fold change in gene expression was calculated using Bio-Rad CFX Manager 2.3 software (Bio-Rad Laboratories, Inc., 2.3, Hercules, CA, USA). The primer sequences are reported in Table A1 in Appendix A.

### 2.6. TNFα Treatment

IPF FBs and HPFs were seeded in 6-well plates at a density of 6000 cells/cm^2^. After 48 h, the fibroblasts were pretreated with R428 (1 and 5 μM) for 1 h and then treated with TNFα (10 ng/mL) for 6 h. Cells were collected for RNA extraction (Nucleozol) and the medium was collected for ELISA measurement.

### 2.7. ELISA Assay: sAxl

In cell culture supernatant soluble Axl (sAxl) was determined in duplicate by ELISA assay using commercial kits (R&D Systems DuoSet Elisa DY008 and DY154, McKinley, MN, USA) following the manufacturer’s instructions. Absorbance was measured using a Victor X4 microplate reader (PerkinElmer, Waltham, MA, USA). Optical density at 450 nm was fitted versus a calibration curve generated with standards, according to the manufacturer’s instructions.

### 2.8. Peripheral Blood Mononuclear Cell (PBMC) Isolation and Differentiation into Macrophages

PBMCs were isolated from human blood using Lymphosep (Biowest) gradient centrifugation according to the manufacturer’s instructions. After counting the PBMCs, 1 × 10^6^ cells/mL were seeded in 6-well plates in RPMI 1640 with 1% penicillin/streptomycin, but without FBS. The cells were allowed to adhere in the 37 °C, 5% CO_2_ incubator for approximately 2 h. Non-adherent cells were removed by washing the wells three times with RPMI 1640 and/or PBS. For the generation of monocyte-derived macrophages (MDMs), the adherent monocytes were cultured with RPMI 1640 media containing 10% FBS, 1% penicillin/streptomycin, M-CSF (10 ng/mL), and GM-CSF (1 ng/mL) for 5 days. Medium was changed every 2–3 days. After differentiation, MDMs were stimulated with either LPS (20 ng/mL) and INF-γ (20 ng/mL) (M1-stimulation), IL-4 (20 ng/mL), IL-10 (20 ng/mL), and IL-13 (20 ng/mL) (M2-stimulation) or left untreated (M0) for 24 h. RNA was extracted, and polarization was confirmed by analyzing the gene expression of M1 and M2 macrophage markers.

### 2.9. Co-Culture: Fibroblasts and Macrophages

MDMs and fibroblasts (IPF FBs and HPFs) were co-cultured using a Transwell system featuring a polyethylene terephthalate 0.4 μm pore size insert membrane. PBMCs were seeded on the lower chamber and were differentiated into macrophages according to the previous protocol (2.8). IPF FBs and HPFs were placed in 6-well plate inserts (6000 cells/cm^2^) in RPMI 1640 (10% FBS, 1% penicillin/streptomycin), and after 72 h, the inserts were transferred to the 6-well plate wells containing the MDM-M0 (day 5). At this point, both chambers had the same medium (RPMI 1640, 10% FBS, 1% penicillin/streptomycin), without any other added stimulant or cytokine. The co-culture was maintained in culture for four days to allow sufficient time for cell-cell signaling. RNA was extracted from macrophages (day 9) to analyze the expression of the M1 and M2 macrophage phenotype markers.

### 2.10. Statistical Analysis

Statistical analyses were performed using GraphPad Prism 6 (GraphPad Software, Inc., La Jolla, CA, USA). Nonparametric Mann–Whitney U-test and Kruskal–Wallis H-test followed by Dunn’s post hoc analysis was used. The data are presented as means ± SEMs.

The significance level was set at *p* < 0.05. Prism ranges of *p*-values are indicated with asterisks: * *p* < 0.05; ** *p* < 0.01; *** *p* < 0.001.

## 3. Results

### 3.1. MTT Assay: Cytotoxicity of TAM Inhibitors, Nintedanib, and TGF-β

**Figure 2:**
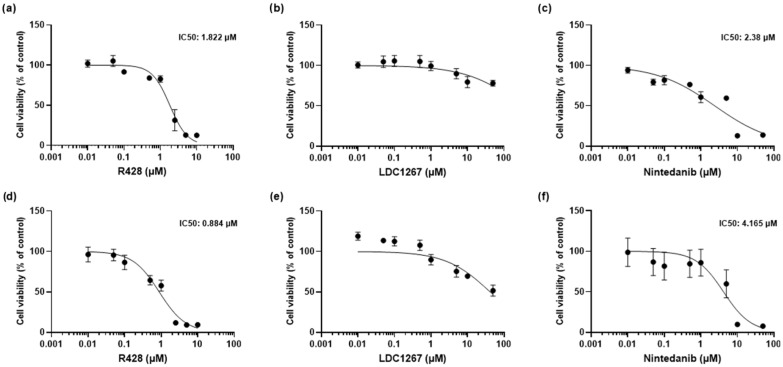
Cell viability and IC_50_ were calculated based on the results of the MTT assay. In the upper panel, cell viability of HPFs is shown, while in the lower panel, IPF FBs were treated with R428 (**a**,**d**), LDC1267 (**b**,**e**), and Nintedanib (**c**,**f**). Data are presented as mean ± SEM values from 3 independent experiments.

**Figure A1:**
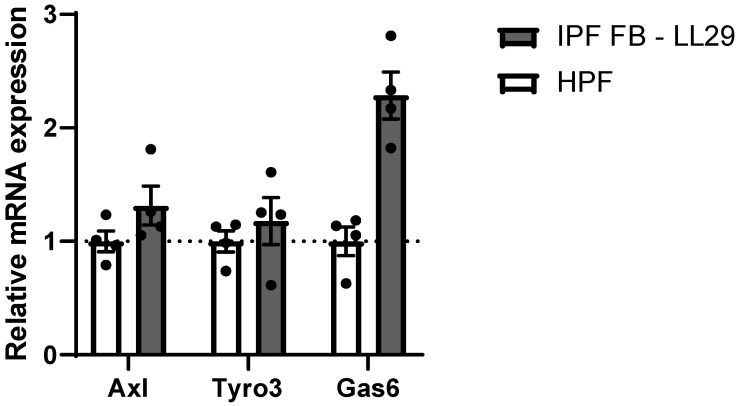
Relative mRNA expression levels of Axl, Tyro3, and Gas6 genes in IPF FBs and HPFs quantified by RT-qPCR. No Mer expression was detected in both fibroblast types. TBP gene was used as housekeeping gene. *N* = 4.

IPF FBs and control HPFs were treated with either R428 (Axl-specific inhibitor), LDC1267 (TAM inhibitor), or Nintedanib at a range of concentrations (from 0.01 to 100 μM) for 48 h. The relative cell viability at each concentration compared to the vehicle control (Figure 2) was determined through the MTT assay. The data from the drug titration curves were analyzed for IC_50_ (except for LDC1267, which presented relatively low toxicity at the studied concentrations). R428 IC_50_ values for IPF FBs and HPFs were approximately 0.88 μM and 1.82 μM, respectively, whereas the IC_50_ for Nintedanib was 4.16 μM in IPF FBs and 2.38 μM in HPFs. In contrast, treatment with LDC1267, even at the highest tested concentration (100 μM), did not reduce the viability below 50% in HPFs and IPF FBs. Overall, IPF FBs seemed to be more sensitive to R428 and more resistant to Nintedanib compared to HPF cells. This can be partly attributed to differential gene expressions of TAM receptors and Gas6 (Figure A1 in Appendix A).

**Figure A2:**
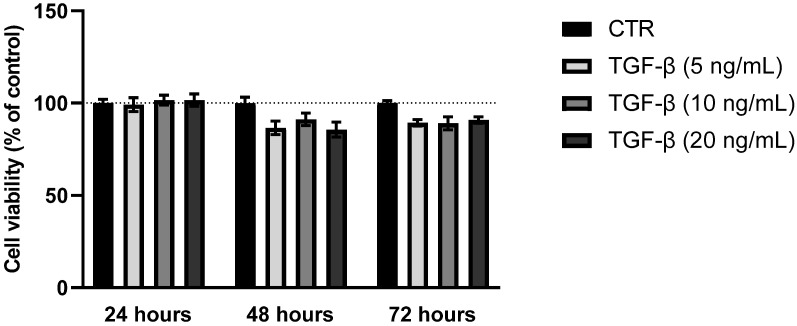
Results of MTT assay evaluating the cell viability of HPFs treated with TGF-β (5, 10, or 20 ng/mL) for 24, 48, and 72 h. *N* = 3.

The cell viability of HPFs was also evaluated by MTT assay after treatment with TGF-β at different concentrations and time-points, as shown in Figure A2 in Appendix A.

The results of the MTT assay revealed that different concentrations of TGF-β (5, 10, or 20 ng/mL) at different time-points (24, 48, and 72 h) did not significantly affect HPF viability. The concentration selected for the following experiment was 10 ng/mL for 48 h of exposure.

### 3.2. TAM Inhibitors: Effect on Cell Proliferation and Cell Migration

**Figure 3:**
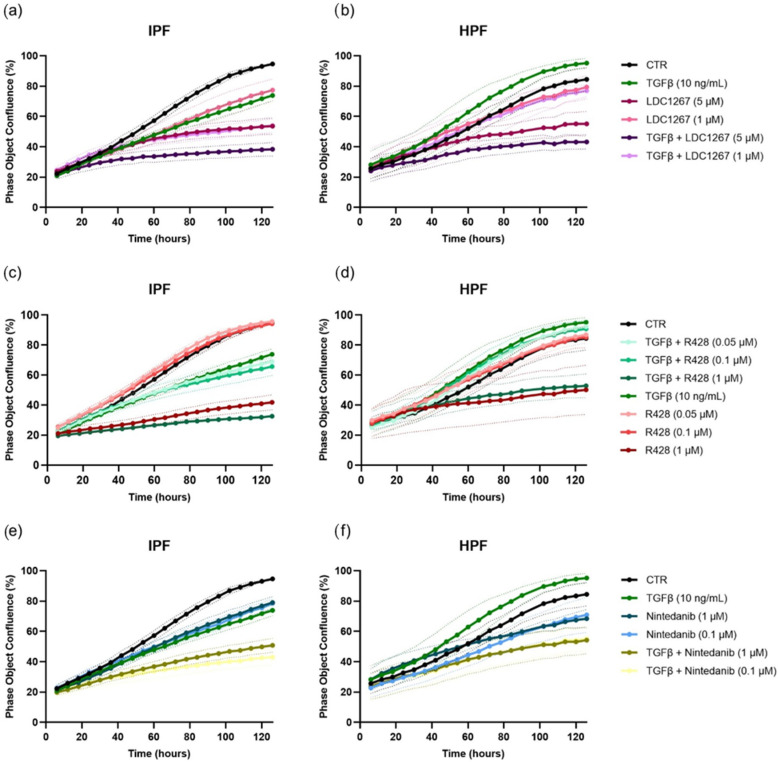
Cell proliferation assay of IPF FBs (**a**,**c**,**e**) and HPFs (**b**,**d**,**f**) treated with different concentrations of LDC1267 (5 μM and 1 μM), R428 (1 μM, 0.1 μM, and 0.05 μM), and Nintedanib (1 μM and 0.1 μM) in the presence or absence of TGF-β (10 ng/mL) was performed using IncuCyte^®^ (Sartorius). Data are presented as mean ± SEM values from 3 independent experiments. SEM is depicted as dashed boundaries.

We determined the effect of TAM targeting approaches in functional assays with both HPFs and IPF FBs. Using the IncuCyte^®^ live cell analysis system (Sartorius), we monitored the proliferation of fibroblasts treated with TGF-β1 (10 ng/mL); the drugs R428, LDC1267, and Nintedanib; and the combination of TGF-β1 and the drug over 126 h (Figure 3). We observed that, in HPFs, TGF-β1 stimulated cell proliferation compared to controls, whereas in IPF FBs, it showed growth-inhibitory effects. In addition, while for LDC1267 and Nintedanib only minor differences in cell confluence were detected between IPF FBs and HPFs, R428 at a higher concentration (1 μM) seemed to have a higher inhibitory impact on IPF FBs. Moreover, the addition of TGF-β1 to the drugs seemed to potentiate the growth-inhibitory effect of TAM inhibitors in IPF FBs.

**Figure 4:**
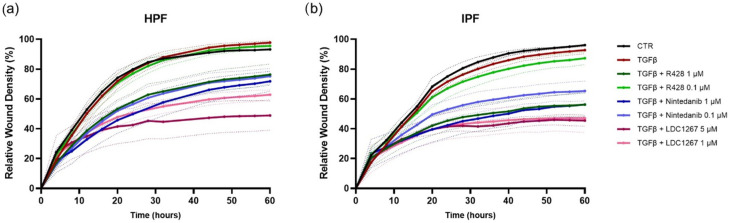
IncuCyte^®^ (Sartorius) scratch wound assay was used to create a wound in a confluent monolayer of HPFs (**a**) or IPF FB (**b**). TGF-β, alone or in combination with different concentrations of R428, Nintedanib, and LDC1267, was added, and the relative wound density (%) was measured every 4 h. Data are presented as mean ± SEM values from 3 independent experiments. SEM is depicted as dashed boundaries.

We also evaluated the capacity of the compounds to inhibit the migration of HPFs and IPF FBs (Figure 4a,b). To do that, we performed a 96-well IncuCyte^®^ scratch wound assay (Sartorius). The different tested treatments included TGF-β1 alone or in combination with different drug concentrations. When the cells were approximately 100% confluent, a scratch was performed in the middle of the well, and the compounds were added in media containing only 1% FBS to minimize the impact of fibroblast growth and migration. Relative wound density (%) was measured every 4 h over 60 h. LDC1267, at both concentrations (5 μM and 1 μM), showed the highest impact on the wound closure. IPF FBs were generally more affected by the different treatments and showed higher delays in wound closure. In particular, the treatment with R428 1 μM led to a relative wound closure of 76% in HPFs, but only 56% in IPF FBs, by the end of the 60 h.

### 3.3. Expression of Fibrotic Genes in TGF-β-Induced Myofibroblasts: Effect of TAM Inhibitors

**Figure 5:**
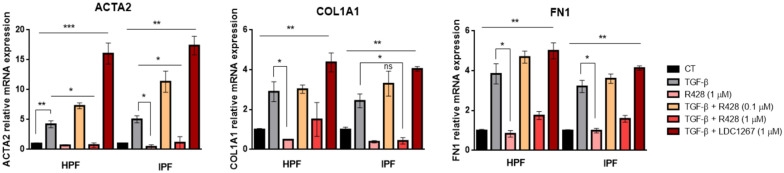
Relative mRNA expression of ACTA2, COL1A1, and FN1 pro-fibrotic genes evaluated by RT-qPCR in HPFs and IPF FBs treated for 48 h with R428 (1 μM) and TGF-β (10 ng/mL), alone or in combination with R428 (0.1 μM), R428 (1 μM), and LDC1267 (1 μM). Data are presented as mean ± SEM values from 3 independent experiments. * *p* < 0.05, ** *p* < 0.01, and *** *p* < 0.001.

To evaluate the activity of TAM inhibitors during fibroblast–myofibroblast transition, HPFs and IPF FBs were treated for 48 h with R428 (1 μM) (concentration that showed relevant results in the proliferation assay; Figure 3c,d) and TGF-β (10 ng/mL), alone or in combination with either R428 (0.1 μM), R428 (1 μM), or LDC1267 (1 μM), and the gene expression of fibrosis-associated markers was evaluated by RT-qPCR (Figure 5). TGF-β treatment markedly upregulated the expression of the ACTA2, COL1A1, and FN1 genes. This effect was further enhanced by co-treatment with R428 (0.1 μM) and LDC1267. While both inhibitors increased the transcript levels of all three fibrotic markers, LDC1267 exerted a more pronounced effect at the tested concentration. Moreover, in TGF-β-treated HPFs, ACTA2 gene expression was significantly higher compared to both R428 (1 μM)-treated group and TGF-β + R428 (1 μM) conditions. TGF-β treatment statistically increased the gene expression of COL1A1 and FN1 when compared to HPFs treated with R428 (1 μM). 

In IPF FBs, TGF-β significantly increased ACTA2, COL1A1, and FN1 expression compared to R428 (1 μM) and TGF-β + R428 (1 μM) conditions. The experimental results are shown in Figure 5.

### 3.4. Effect of Axl Targeting on TNFα-Induced IL-6 and TGF-β Expression

**Figure 6:**
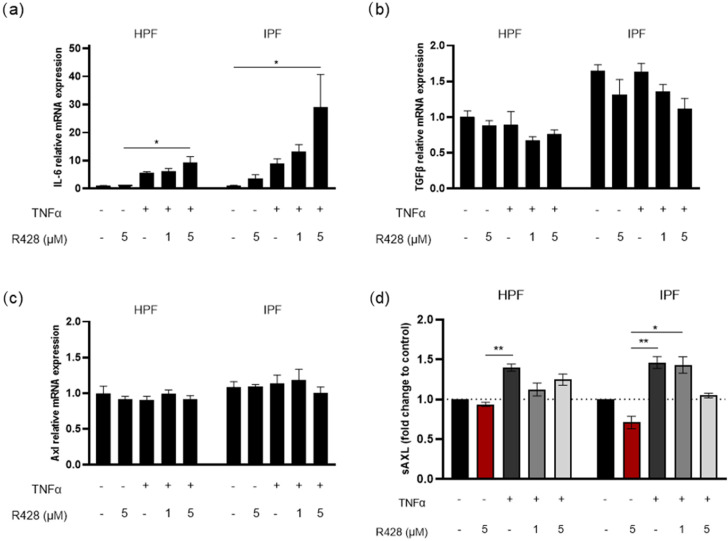
Influence of R428 treatment on IL-6, TGF-β, and Axl in IPF FBs and HPFs. IPF FBs and HPFs were pre-incubated with R428 (1 μM or 5 μM) for 1 h and then incubated with TNFα (10 ng/mL) for 6 h. Total RNA was isolated and IL-6 (**a**), TGF-β (**b**), and Axl (**c**) mRNA levels were measured by RT-qPCR. ELISA assay was used to measure the levels of sAxl (**d**) in the supernatant. Data are presented as mean ± SEM values from 4 independent experiments. * *p* < 0.05; ** *p* < 0.01.

The role of Axl in TNFα-induced gene expression was examined by pretreating IPF FBs and HPF cells with R428 (1 μM and 5 μM) for 1 h, followed by stimulation with TNFα (10 ng/mL) for 6 h. The mRNA was extracted for the evaluation of gene expression by RT-qPCR, and the conditioned medium was collected for the measurement of released sAxl by ELISA. On one hand, TNFα stimulation led to increased IL-6 expression, and pretreatment with R428 seemed to have a synergistic effect enhancing IL-6 levels (Figure 6a). On the other hand, TGF-β expression was not induced by TNFα under the tested conditions; however, TGF-β expression was decreased by R428 pretreatment, particularly in IPF FBs (Figure 6b). Of note, the expression of TGF-β at control conditions was higher in IPF FBs compared to HPF. 

Axl transcription was evaluated to verify the impact of TNFα treatment and Axl-inhibitor pretreatment on Axl levels (Figure 6c). No significant differences were observed across conditions and cell types. However, the analysis of sAxl levels in the conditioned media revealed that both IPF FBs and HPF cells released higher amounts of sAxl in response to TNFα, which was moderately reduced by R428 pretreatment (Figure 6d).

### 3.5. Macrophage Polarization: Fibroblast-Mediated Effects

**Figure 7:**
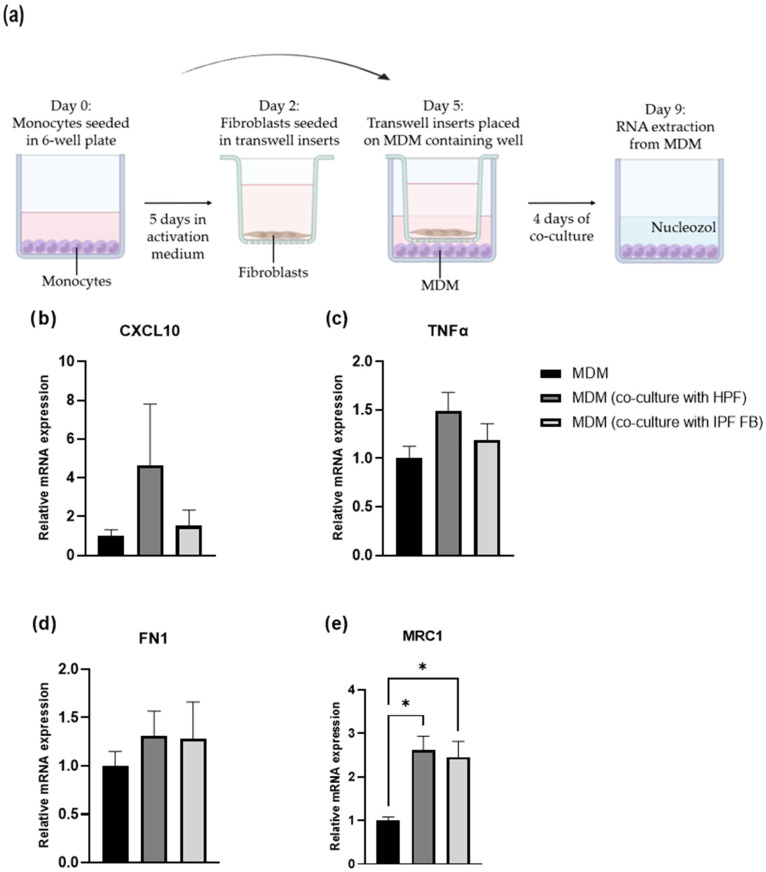
Effects of HPFs and IPF FBs on MDM. M0 MDM wereMDMs were co-cultured with HPFs or IPF FBs using a Transwell system for 4 days (**a**). Following co-culture, RNA was extracted from MDMs, and gene expression of M1 (**b**,**c**) and M2 (**d**,**e**) polarization markers was evaluated by RT-qPCR. Data are presented as mean ± SEM values from 4 independent experiments. * *p* < 0.05.

**Figure A3:**
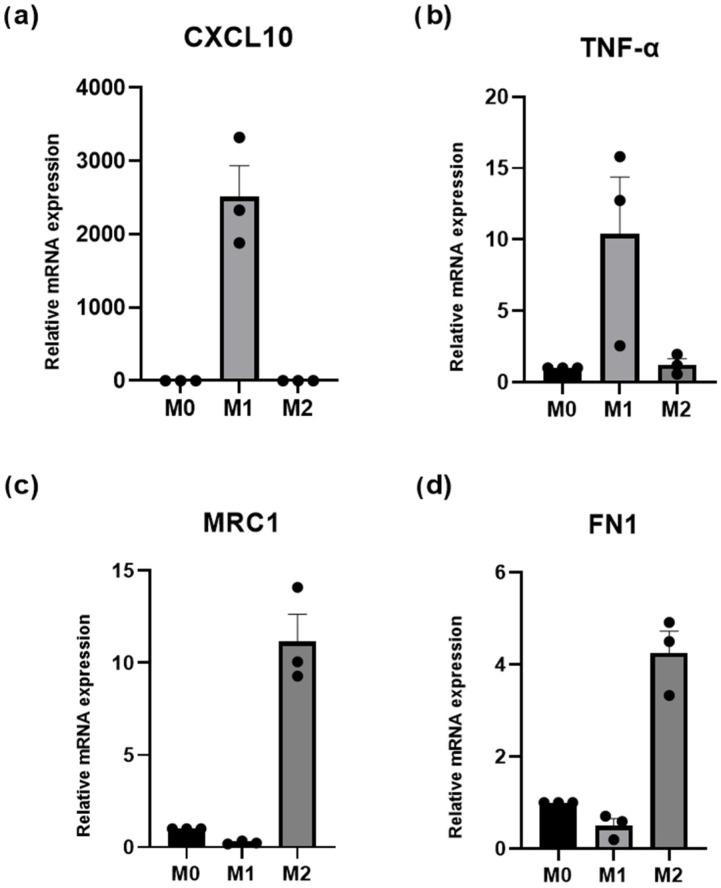
Quantification of M1 (**a**,**b**) and M2 (**c**,**d**) markers in M0, M1, and M2 macrophages by RT-qPCR. RNA expression data was normalized to HPRT. *N* = 3.

To investigate the effects of HPFs or IPF FBs on MDM polarization, we established a co-culture Transwell system with non-polarized MDMs (M0) and the control or IPF-derived fibroblasts (Figure 7a). MDMs and fibroblasts were separated by a 0.4 μm pore membrane to allow indirect communication through soluble factors for a period of 4 days. Thereafter, we analyzed the expression of specific M1 and M2 markers in the MDMs (Figure A3 in Appendix A) by RT-qPCR. The results presented in Figure 7b–e represent the mean expression from four different healthy MDM donors. Co-culture with both HPFs and IPF FBs significantly increased MRC1 expression (M2 marker) while both FN1 (also M2-associated) as well as TNFα and CXCL10 (M1 markers) were moderately upregulated.

## 4. Discussion

Idiopathic Pulmonary Fibrosis (IPF) is a progressive lung disease of unknown origin characterized by the fibrosis of lung tissue [28]. It is one of the most aggressive forms of IIPs, the signature of which is a persistent and progressive fibrosis due to abnormal remodeling and collagen deposition [29].

Historically, the high mortality rate and limited treatment options for IPF patients were attributed to the lack of early diagnostic and prognostic tools [30]. Developing effective strategies for early diagnosis is crucial to reduce mortality. A deeper insight on disease mechanisms might allow the identification of reliable biomarkers for accurate diagnosis and prognosis and the development of targeted therapies [29].

While there is currently no cure for IPF, two antifibrotic drugs, Nintedanib and pirfenidone, have been shown to slow disease progression, reduce acute exacerbations [31], and improve mortality rates [32].

In the present paper, we investigated the potential role of the Gas6/TAM system and TAM receptor inhibitors in the pathophysiology of IPF. We focused on the activity of Bemcentinib (R428), a selective Axl inhibitor, and LDC1267, a TAM receptor inhibitor, comparing their effects with those of Nintedanib.

Our findings showed that IPF FBs were more sensitive to R428 and more resistant to Nintedanib when compared to HPFs (Figure 2a–f). This sensitivity to R428 may be attributed to the potent inhibitory activity of the drug on Axl, even at lower concentrations [33]. The differential gene expression of TAM receptors and Gas6 in the two cell lines, as shown in Figure A1 in Appendix A, may also contribute to these results. Indeed, higher TAMs and Gas6 expression levels seem to be detected in IPF FBs compared to normal HPF cells, consistently with the finding of Espindola et al. [26].

We also assessed the cytotoxicity of TGF-β, a pro-fibrotic cytokine involved in many fibrotic diseases such as pulmonary fibrosis [34,35,36]. High concentrations of TGF-β and prolonged incubations induce the differentiation of fibroblasts into myofibroblasts, leading to the deposition of the extracellular matrix [37].

We also evaluated HPF and IPF FB proliferation, assessing the effect of R428, LDC1267, and Nintedanib at predetermined concentrations, both in the presence and absence of TGF-β (Figure 3). While the effects of LDC1267 and Nintedanib were similar between both cell types, R428 (1 μM) showed a higher inhibitory effect on the confluence of IPF FBs. Indeed, R428 resulted in reduced fibroblast proliferation, suggesting its potential as an antifibrotic treatment agent in IPF [26].

Moreover, in healthy lung fibroblasts TGF-β seems to be able to stimulate the release of fibroblast growth factor 2 (FGF-2), which, in turn, can activate p38 MAPK e JNK signaling pathways [38], as well as FGF-2/ERK [39] and circ_PWWP2A-mediated pathways [40], resulting in increased proliferation. Conversely, in IPF lung FBs, TGF-β stimulation seems to promote a characteristic senescent phenotype inducing endoplasmic reticulum (ER) stress consequently involved in the mitochondrial dysfunction and cellular senescence reducing proliferation [41].

In addition, we evaluated the effect of TAM inhibitors and Nintedanib in the presence or absence of TGF-β on HPF and IPF FB migration using a scratch assay [42,43]. LDC1267, at both concentrations (5 μM and 1 μM), showed the most significant impact on wound closure, with IPF FBs exhibiting delayed wound closure (Figure 4). Our results are consistent with previous studies showing R428 efficacy in arresting IPF FB migration compared to other Gas6/TAM targeting strategies [26].

The expression of fibrosis-associated markers ACTA2, COL1A1, and FN1 was assessed in HPFs and IPF FBs. R428 showed a dose-dependent response, with a lower concentration having a synergistic effect with TGF-β, while a higher concentration (1 μM) significantly reduced their gene expression (Figure 5). Direct investigations into the specific roles of R428 and LDC1267 in modulating the expression of ACTA2, COL1A1, and FN1 in IPF disease are limited; nevertheless, in line with our results, Steiner et al. (2021) evaluated the role of R428 on human colonic fibroblasts previously treated with TGF-β [44]. However, substantial evidence exists in the literature regarding the involvement TGF-β in the upregulation of these fibrotic markers [45], supporting its pivotal role in fibrogenesis.

We further investigated the role of Axl in TNFα-induced IL-6 and TGF-β gene expression in HPFs and IPF FBs. TNFα is a potent pro-inflammatory cytokine involved in several pulmonary disorders such as IPF [46]; it led to an increase in IL-6 expression in both cell types. This finding was in accordance with previous evidence [47,48]. Increased levels of IL-6 have been also associated with a poorer outcome in IPF patients [49], underlining its potential as an emerging target strategy to treat lung fibrosis [50,51]. R428 seems to upregulate IL-6 expression (Figure 6a); however, the literature lacks in vitro studies evaluating the direct effects of the compounds on this cytokine. Nevertheless, in bleomycin-induced pulmonary fibrosis mice models, Axl deficiency leads to reduced IL-6 and TNFα expression and a decrease in M2-like macrophage differentiation in lung tissue [52]. Furthermore, pharmacological Axl inhibition attenuated fibrotic markers in smoking-related pulmonary fibrosis, indicating an anti-inflammatory effect [53]. Regarding TGF-β, our findings differed from those of previous studies, as we did not observe upregulation after TNFα treatment [54]. However, R428 pretreatment decreases TGF-β expression, especially in IPF FBs (Figure 6b). Of note, TGF-β expression was higher in IPF FBs compared to HPFs. In bleomycin-induced pulmonary fibrosis mice models, pharmacological Axl inhibition contributes to antifibrotic processes reducing factors associated with fibroblast differentiation and fibrosis [55]. Although TNFα did not affect Axl gene expression in HPFs and IPF FBs, we observed an increase in sAxl levels in the culture medium (Figure 6c,d), likely due to ectodomain shedding mediated by ADAM10/17 proteases, in inflammatory contexts [56]. This increase was partially reversed by R428.

Macrophages are essential cells of the innate immune system responsible for phagocytosis and pathogen elimination. Their polarization, influenced by cytokines, chemokines, and transcription factors, plays a critical role in inflammatory disorders like lung diseases [57], including IPF. In IPF, macrophage polarization status affects disease progression. Indeed, to modulate the fibrotic response, a balance between M1 and M2 phenotypes is crucial [58]. Considering their central involvement in the pathogenesis of IPF, we conducted experiments to study the interactions between macrophages and pulmonary fibroblasts potentially impacting disease development and progression. In vitro, the stimulation with INF-γ and LPS upregulated the expression of CXCL10 and TNFα genes, typical of the M1 pro-inflammatory phenotype [59]. Conversely, the stimulation with IL-4, IL-10, and IL-13 leads to an M2 anti-inflammatory phenotype [60] and the upregulation of MRC1 and FN1 genes (Figure A3 in Appendix A).

The co-culture experiment (Figure 7a) showed a significantly increased expression of MRC1 (M2 marker) in the presence of both fibroblast types while FN1 (M2 marker) and TNFα, CXCL10 (M1 markers) were moderately increased, indicating fibroblast influence on macrophage polarization (Figure 7b–e). However, no significant difference was observed between HPF and IPF FB co-cultures. In a co-culture model of vocal fold fibroblasts and macrophages, the upregulation of FN1, together with TGM2 and LOX (ECM-crosslinking enzymes), has already been reported [61], suggesting that fibroblast-derived signaling promotes pro-fibrotic rather than inflammatory macrophage responses. Similarly, Holt et al. demonstrated that conditioned media from un-activated RAW 264.7 and bone-marrow-derived macrophages transiently increased pro-inflammatory cytokine secretion (IL-6, TNFα, MCP-1, MIP-1α/β) in NIH 3T3 fibroblasts, primarily within the first 3 days. Conversely, fibroblast-conditioned media suppressed cytokine production in macrophages, suggesting a negative feedback loop between these cells to limit inflammation [62]. In co-cultures of synovial fibroblasts and differentiated macrophages, synovial fibroblasts inhibited TNF-driven IFN-β autocrine signaling and downstream IFN-stimulated gene expressions such as of CXCL10 and CXCL9, thereby reducing inflammation [63]. Yadav and colleagues recently identified a paracrine circuit between macrophages and fibroblasts [64], revealing a bidirectional macrophage–fibroblast crosstalk where macrophages induce IL-6 expression in fibroblasts leading to increased arginase 1 (Arg1) expression in macrophages. In activated fibroblasts, Arg1 appears to modulate collagen production through ornithine metabolism. The interaction between fibroblasts and alveolar macrophages in the IPF context was also investigated in a separate study [65]. The direct co-culture of normal or IPF alveolar macrophages induced distinct changes in gene expression and fibroblast contraction in normal or IPF lung fibroblasts.

**Figure A4:**
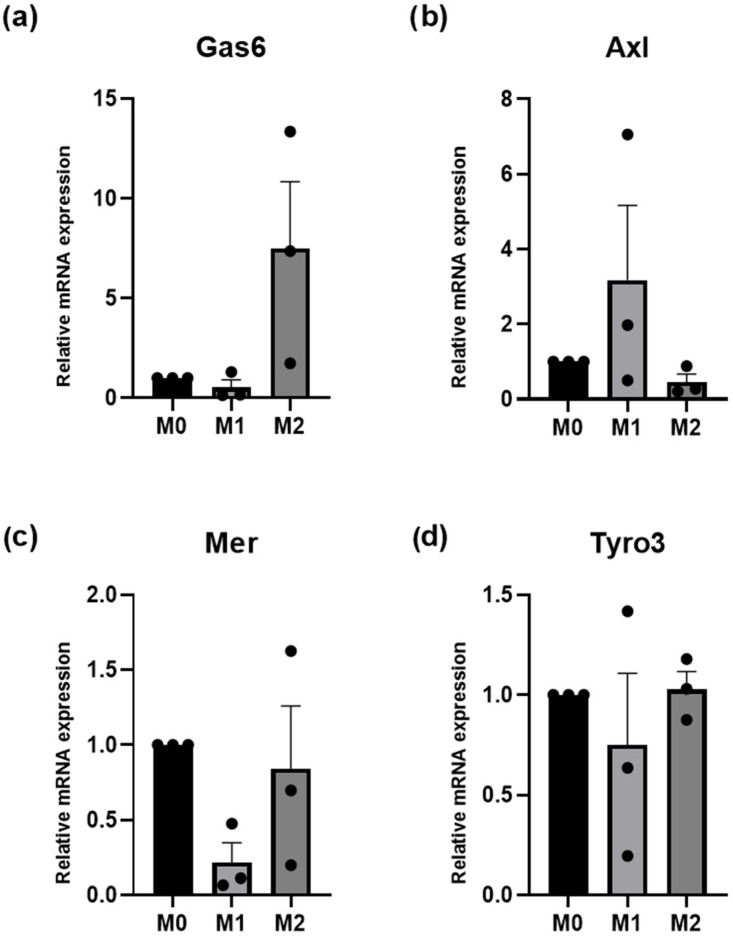
Quantification of (**a**) Gas6, (**b**) Axl, (**c**) Mer, and (**d**) Tyro3 expression in M0, M1, and M2 macrophages by RT-qPCR. RNA expression data was normalized to HPRT. *N* = 3.

In co-culture experiments, we assessed the impact of HPFs–IPF FBs on MDM. Future studies will focus on the influence of polarized macrophages, particularly M2 MDMs, on fibrotic markers of fibroblasts and the effects of small-molecule TAM inhibitors. Indeed, probably, different effects on gene modulation may be observed also because M2 MDMs express more Gas6 compared to the other phenotypes as shown in Figure A4 in Appendix A. Previous studies have shown elevated Gas6 expression in murine M2 macrophages compared to the M1 phenotype [66]. However, species-specific differences between murine and human macrophages limit the direct translation of these findings [67,68]. Further research on the Gas6/TAMs axis in IPF development is warranted as M2-like macrophages are dominant in later stages of fibrosis, secreting pro-fibrotic mediators such as TGF-β1 and platelet-derived growth factor (PDGF) that promote fibroblast activation and collagen deposition [69].

## 5. Conclusions

Our findings suggest that R428 and LDC1267 affect the proliferation, migration, and gene expression of activated fibroblasts, potentially influencing the fibrotic process by modulating TAM signaling. Co-culture experiments demonstrate that fibroblasts can impact macrophage polarization, emphasizing the significance of cell-to-cell interactions in fibrotic pathologies. These findings potentially pave the way for further studies assessing the effectiveness of Gas6/TAM targeting strategies in the treatment of IPF.

## Data Availability

Data generated or analyzed during this study can be available after specific request.

## Abbreviations

The following abbreviations have been used in this manuscript:

TAMs	Tyro3, Axl, and Mer receptors
Gas6	Growth Arrest-Specific 6
IPF	Idiopathic Pulmonary Fibrosis
HPF	Human pulmonary fibroblast
IPF FBs	IPF fibroblasts
R428	Bemcentinib (Axl-specific inhibitor)
LDC1267	TAM inhibitor
TGF-β	Transforming Growth Factor Beta
ACTA2	Actin Alpha 2, Smooth Muscle
FN1	Fibronectin 1
COL1A1	Collagen Type I Alpha 1 Chain
MRC1	Mannose Receptor C-Type 1
TNFα	Tumor Necrosis Factor Alpha
CXCL10	C-X-C Motif Chemokine Ligand 10
ILDs	Interstitial Lung Diseases
UIP	Usual Interstitial Pneumonia
IIPs	Idiopathic Interstitial Pneumonias
AEC2s	Type 2 alveolar epithelial cells
AEC1s	Type 1 alveolar epithelial cells
EMT	Epithelial–Mesenchymal Transition
RTKs	tyrosine kinases receptors
ProS1	Protein S
sTyro3	soluble Tyro3
sAxl	soluble Axl
sMer	soluble Mer
FBS	Fetal Bovine Serum
DMSO	Dimethyl sulfoxide
PBS	Phosphate Buffered Saline
NTC	No template control
NRT	No reverse transcriptase control
HPRT	Hypoxanthine Phosphoribosyltransferase 1
TBP	TATA-Box Binding Protein
PBMC	Peripheral Blood Mononuclear Cell
M-CSF	Macrophage Colony-Stimulating Factor
GM-CSF	Granulocyte–Macrophage Colony-Stimulating Factor
MDM	Monocyte-derived macrophage
LPS	Lipopolysaccharide
IFN-γ	Interferon-γ
IL-4	Interleukin 4
IL-10	Interleukin 10
IL-13	Interleukin 13
IC_50_	Inhibitory Concentration 50
SEM	Standard Error of the Mean
IL-6	Interleukin 6
FDA	Food and Drug Administration
α-SMA	α-smooth muscle actin
FGF-2	Fibroblast Growth Factor 2
ADAM10/17	A Disintegrin And Metalloproteinase Domain 10/17
TGM2	Transglutaminase-2
LOX	Lysyl oxidase
ECM	Extracellular matrix
Arg1	Arginase 1
MCP-1	Monocyte Chemoattractant Protein-1
MIP-1α/β	Macrophage Inflammatory Protein 1α/β
CXCL9	C-X-C Motif Chemokine Ligand 9
PDGF	platelet-derived growth factor

## Appendix A

**Table A1:** This table contains the genes of interest and their specific primer (Forward and Reverse) sequences used to determine their expression through RT-qPCR.

Genes	Forward	Reverse
Axl	GGTGGCTGTGAAGACGATGA	CTCAGATACTCCATGCCACT
Mer	GCCCCATCAGTAGCACCTTT	TGCACGTAGCATTGTGGACT
Tyro3	CAATGCAGTTGGCTGTGGAC	CGTTAGCACACCAAGGACCA
Gas6	ACGACCCCGAGACGGATTAT	CTTCCTATCGCAGGGGTTGG
ACTA2	AGCCAAGCACTGTCAGGAAT	TTGTCACACACCAAGGCAGT
COL1A1	CAGGCTGGTGTGATGGGATT	GGGCCTTGTTCACCTCTCTC
FN1	GGGTCTCCTCCCAGAGAAGT	GGAAGGGTTACCAGTTGGGG
IL-6	TCACTGGTCTTTTGGAGTTTGA	AGAGCCCTCAGGCTGGACT
TFG-β	CCCAGCATCTGCAAAGCTC	GTCAATGTACAGCTGCCGCA
CXCL10	GTGGATGTTCTGACCCTGCT	GGAGGATGGCAGTGGAAGTC
TNF-α	CCCAGGGACCTCTCTCTAATC	ATGGGCTACAGGCTTGTCACT
MRC1	ACCTGCGACAGTAAACGAGG	TGTCTCCGCTTCATGCCATT
HPRT	GATTTGGAAAGGGTGTTTAT	TCCCATCTCCTTCATCACAT
TBP	TGTATCCACAGTGAATCTTGGTTG	GGTTCGTGGCTCTCTTATCCTC
